# Behavioral Implications of the Complete Absence of Guests on a Zoo-Housed Gorilla Troop

**DOI:** 10.3390/ani11051346

**Published:** 2021-05-09

**Authors:** Megan E. Miller, Caeley M. Robinson, Susan W. Margulis

**Affiliations:** 1 Department of Animal Behavior, Ecology, and Conservation, Canisius College, Buffalo, NY 14208, USA; mille254@canisius.edu (M.E.M.); robins97@canisius.edu (C.M.R.); 2 Department of Biology, Canisius College, Buffalo, NY 14208, USA

**Keywords:** gorilla, visitor effects, activity budget

## Abstract

**Simple Summary:**

The welfare of animals in human care should be taken into consideration by both the scientific community and members of the public who are able to view animals in a zoo setting. One component of individual welfare is how groups of animals and the individuals within groups may be affected by the presence of zoo guests. At the Buffalo Zoo in New York, we studied the behavioral differences of six gorillas in two conditions: heavy guest presence and the complete absence of guests. We found few significant behavioral differences between the conditions, with the greatest behavioral change observed in the adult silverback male. We stress the importance of not over-generalizing our findings, and suggest an inter-institutional study of the nuanced ways that zoo guests interact with gorillas in managed care.

**Abstract:**

Research conducted on the effects that zoo visitors have on primate behavior has yielded inconsistent patterns. This study aims to contribute to the growing body of literature regarding visitor effects on zoo-housed primate’s activity budgets, with the purpose of quantifying the behavioral variability under two conditions: guest presence and guest absence. Due to the COVID-19 pandemic, many zoos were closed to the public for varying lengths of time. The Buffalo Zoo was closed to guests for an 18-week period including the summer of 2020, which allowed us to effectively control for zoo guest presence. This case report compares data on the zoo’s gorilla troop from the same time period in 2019. We found inconsistent results, similar to prior studies conducted with zoo-housed gorilla troops. Most gorillas were observed foraging less and exhibiting more inactivity in 2020, whereas the adult male silverback showed the opposite pattern. Abnormal or undesirable behaviors were performed less frequently when guests were absent however, these differences were not significant. We encourage others to compare behavior patterns during the pandemic shutdown to add to our knowledge base of visitor effects. We suggest that researchers do not try and generalize their individual and troop results to the entire population of gorillas in managed care, as both intrinsic and extrinsic factors contribute to individual differences in behavioral response.

## 1. Introduction

The welfare of animals in managed care has received considerable attention in recent years [1,2,3]. One condition that may influence welfare is the presence of zoo guests and the unpredictable variability that zoo guests bring to exhibit spaces [4]. Investigating how visitors may or may not influence animal behavior in a zoo setting can help institutions better understand how the environment affects the animals’ welfare. Zoos can use this information to adapt husbandry practices such as the amount of time animals spend on exhibit or how exhibits are designed. It can also help provide a deeper comprehension of general human–animal relationships.

Each individual animal is likely to express its own idiosyncratic and variable responses to zoo guests, which is why the study of the zoo visitor effect [4,5] is important in quantifying visitor impacts to animal behaviors. The zoo visitor effect shows that species—and individuals within each species—may express varying welfare impacts from long-term exposure to unfamiliar and unpredictable zoo visitors. For some species, visitors can be viewed as positive and novel enrichment [4]. However, for other species across a plethora of taxa, including harbor seals (*Phoca vitulina*), koalas (*Phascolarctos cinereus*), and hornbills (*Bucorvus abyssinicus*), the presence of visitors can lead to negative welfare impacts that elicit stereotypic behaviors and even prolonged stress in individuals [6,7,8]. These studies often provide the baseline knowledge for the inception of new and improved welfare standards in zoos.

Primates have been particularly well-studied with respect to possible impacts of zoo visitors on individuals and intra-troop interactions. Black-capped capuchins (*Sapajus apella*) have shown a decrease in aggression and abnormal behaviors when viewing windows of their exhibit were obstructed [9]. Although not in a zoo setting, chimpanzees in a sanctuary open to visitors were found to locomote more when visitors were given access to viewing areas, though they showed inter-individual differences in reactions to sanctuary visitors [10,11]. Visitors were more likely to influence orangutan (*Pongo spp*) behavior when crowds were large, visitors were close to the individual orangutans, and when visitors possessed food [12]. White-handed gibbons (*Hylobates lar*) were more likely to engage in territorial displays (via song or increased brachiation), self-scratching behaviors, and social bonding behaviors such as allogrooming in periods of high noise levels and large crowds [13].

The impacts of zoo visitors are well-documented in Western lowland gorillas (*Gorilla gorilla gorilla*) as well, and these zoo-housed troops have been shown to exhibit variable responses to zoo guests. Their responses tend to show considerable individual variation independent of factors such as age, sex, or rearing history. In a study of four gorilla troops comprising both family and bachelor groups, no troop consistently performed undesirable or abnormal behaviors when crowd levels were high [14]. They also found that sex, age, and rearing history were not determinants of behavioral responses; however, personality factors may have had an influence on individuals’ crowd responses.

In two studies where visual barriers were installed to block direct view with zoo visitors, individuals were less likely to engage in intra-troop aggression, visitor directed aggression, and stereotypy; however, no significant, patterned changes in the gorillas’ activity budgets attributed to guest presence were found in either study [15,16].

High crowd levels have been associated with a decrease in foraging behavior [17,18] and an increase in inactivity and resting behaviors [19,20,21]. Undesirable behaviors, such as overgrooming, plucking, regurgitation and reingestion (R/R), and pacing can increase with denser crowds [19,22,23,24]. When installing a privacy screen on an exhibit window, one study found that a single gorilla ceased to engage in R/R behaviors altogether [17]. Although inconsistent, aggression towards visitors and intra-troop contact and non-contact aggression have been shown to increase with high crowd levels [17,19].

The goal of this study is to corroborate the findings of similar studies that attempt to quantify the effect of the presence of zoo visitors on zoo-housed Western lowland gorillas (*Gorilla*, *gorilla*, *gorilla*). We collected behavioral data on one gorilla troop at the Buffalo Zoo consisting of six individuals. Beginning March 14th of 2020, the novel coronavirus pandemic presented us with a unique experimental condition which removed guests from the gorilla’s environment for a three month period. With no additional external changes to the gorilla’s environment, zoo staffing, daily routines, or any changes within the troop, we opportunistically compared these two conditions, with the only substantive change being the presence or absence of visitors. Thus, this case study allows us to compare the data collected during the pandemic shut-down to our archived data from the same period in 2019 when visitors were present.

## 2. Materials and Methods

### 2.1. Study Site

The study was conducted at the gorilla exhibit at the Buffalo Zoo in Buffalo, NY, USA. All the subjects were housed in an indoor exhibit measuring approximately 185 m^2^ containing a climbing structure and alcoves that allowed the gorillas to be out of view from visitors and observers if they chose. Visitors to the zoo were able to view the gorillas’ exhibit on days that the zoo was open between 10:00 a.m.–4:00 p.m. Four glass viewing windows permitted visual access. The exhibit included two alcoves that led to the off-exhibit holding area. The gorillas had access to the alcoves at all times, and could not be seen by visitors when in this location. During the period of closure in 2020, the gorillas often had access to their off-exhibit holding areas, though access varied periodically. Normal routines continued for feeding and provision of enrichment.

### 2.2. Subjects

We have been collecting behavioral data since 2009 on the group of western lowland gorillas (*Gorilla gorilla gorilla*) housed at the Buffalo Zoo. The family group includes a mature adult male named Koga (hereafter designed as adult male, “AM”), two adult females named Sidney (adult female 1, “AF1”) and Lily (“AF2”), and three offspring: a sub-adult female named Amari (subadult female, “SAF”), a juvenile female named Nyah (juvenile female, “JF”) and a juvenile male named Kayin (juvenile male, “JM”) (Table 1).

### 2.3. Procedures

Behavioral data were collected using a standardized ethogram by members of the Margulis Lab research team at Canisius College in Buffalo, NY. In 2019, we collected data in one or two sessions each day in the morning and/or afternoon during the zoo’s open hours. We collected data from three to seven days each week throughout the year based on availability of student researchers. We conducted twenty-minute instantaneous focal observations [25] on each of the six gorillas in the exhibit, randomizing the order in which we observed each individual. Every minute, the focal subject’s behavior, location, and neighbors were entered into a spreadsheet using the Excel^TM^ app. For the purposes of this investigation, we focus only on selected state behaviors (Table 2). We omitted behaviors that occurred too rarely for analysis unless they were particularly relevant to the study. The behaviors we included in analyses were locomotion, inactivity, forage, self-care, regurgitation/reingestion, pluck, and social play. We also evaluated time spent out of view.

For the 2020 data, the zoo was closed to the public from 14 March through 1 July. One of the authors (SWM) was given access to the zoo’s remote cameras to permit observations during a portion of the closed period. All methods of data collection were kept consistent with in-person observations. These data were collected between 26 May 2020 and 1 July 2020 and comprised 36 h of data.

We used data collected during the summer of 2019, from 27 May 2019 through 2 Sept 2019 (the “busy” summer season) as our comparison. These data provided approximately the same amount of data on each subject (Table 1).

### 2.4. Data Analysis

Before conducting behavioral analyses, we first removed “out of view” observations from the raw data. This included fully removing all observations where the individual was out of view for more than half (11 or more scans out of a possible 20) of the total scans recorded during that session, and including only scans where the animal was visible. All remaining occurrences of an individual being out of view were removed from analyses. There were no significant differences amongst individuals or between the two years in percent time spent out of view.

Data were analyzed in R Studio [26], version 4.0.3. To analyze behavioral change between the two conditions, we conducted Mann–Whitney U Tests using the Bonferroni correction to account for multiple comparisons. *p*-values were adjusted accordingly.

## 3. Results

**Figure 1:**
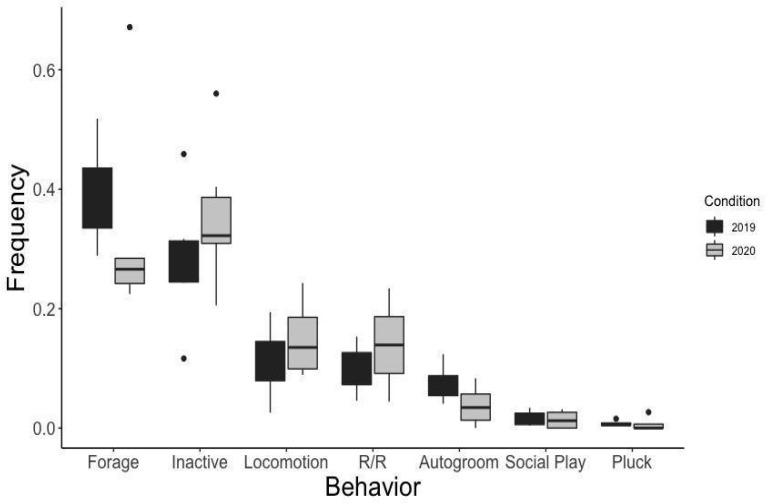
Proportion of time spent in each of the seven analyzed behaviors for all gorillas (n = 6). Box plots indicate the mean, quartiles, and outliers for each behavior.

When data on all six gorillas were combined to give us a whole-troop comparison, we found no significant differences in behavior between the two study periods (Figure 1). We then broke analyses down to study potential individual differences in behavior, and found few significant differences individually. Some non-significant but notable differences were observed in particular behaviors, including foraging, inactivity, locomotion, and autogrooming behaviors. While visitors were absent, five of the six gorillas showed a decrease in foraging with concurrent increases in inactivity. The AM showed opposing patterns, nearly doubling his foraging time and showing notable decreases in inactivity when zoo guests were not in attendance.

**Figure 2:**
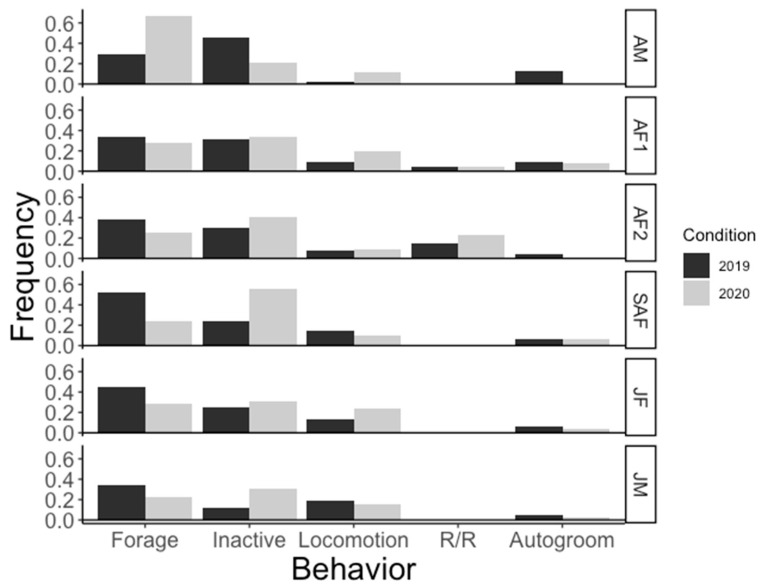
Proportion of time spent in each behavior for each individual gorilla. “Pluck” and “Social Play” were omitted as they occurred too rarely to visualize on the graph.

Mann–Whitney U tests showed very little statistical difference in behaviors for all six study subjects (Table 3; Figure 2). The AM demonstrated the most notable changes in behavior, however only autogroom behavior decreased significantly between the two conditions (U = 99, *p* = 0.0095), comprising 12 percent of his activity budget in 2019 and not being observed at all in the 2020 condition. The SAF’s inactivity level changed significantly (U = 42, *p* = 0.01), more than doubling between the conditions to a total of 56 percent of her activity budget in 2020.

There were also downward trends in foraging behavior observed in five of the six gorillas in the troop, with as much as a 27.8% decrease in the behavior between the conditions (Figure 2). Conversely, the AM’s foraging behavior increased from 28.86% of his activity budget in 2019 to 67.12% of his 2020 activity budget.

Other noted trends in the activity budgets included an increase in inactivity during the 2020 condition for five of the gorillas, with a simultaneous increase in locomotor behavior shown by four of the gorillas. It is important to note that although these changes trended similarly for a majority of the subjects, these changes did not show statistical significance.

All six gorillas had downward trends in autogrooming between the two conditions. Three of four gorillas who were observed plucking in 2019 did not pluck in 2020, however, it is important to note that this behavior occurred infrequently, with each gorilla plucking less than five times in the 2019 condition. The SAF plucked at a much higher rate in 2020, increasing from 0.45% in 2019 to 2.67% of the total activity budget in 2020.

Regurgitation and reingestion was observed in both adult female gorillas in each of the conditions. AF1 exhibited no change in the frequency of performing R/R, while for AF2 R/R comprised 23.40% of her total activity budget in 2020, up from 15.31% in the condition with visitor presence.

Differences in social play were marginal and did not reflect a significant change in the behavior between the two conditions.

## 4. Discussion

The findings of this case report suggest no significant differences in the subjects’ activity budgets between the two conditions. Our findings were consistent with a number of studies that examined visitor effects on gorillas. While we observed some variation in whole-troop behavior, any behavioral changes that were significant at the individual level were not consistent throughout the troop.

We observed slight overall differences in foraging, inactivity, and locomotor behaviors. Foraging behavior decreased in five of the six subjects when visitors were not in attendance; this is contradictory to several previous studies that observed a decrease in foraging when crowd sizes increased in zoo-housed gorillas [17] and chimpanzees [18], respectively. We also observed a general pattern of increased inactivity when visitors were absent, consistent with several other studies [19,21].

It has been suggested that abnormal or undesirable behaviors occur less frequently in primates when crowd levels are low [15,23]. We observed similar trends in autogrooming behaviors, with modest declines of the behavior seen in five of six gorillas, and the AM ceasing to perform the behavior all together. Similar patterns have been observed in other studies [14]; however, the authors prefaced this by stating that any changes in abnormal behaviors were not consistent. We also found inconsistency in the occurrence of abnormal behaviors, most notably with regurgitation and reingestion. While AF1 did not change her rate of performance of this behavior, AF2 engaged in regurgitation and reingestion at nearly double the 2019 rate. Despite this large increase, the change was not statistically significant.

Overall, our findings corroborate what the literature suggests: individual variability in gorilla response to visitor presence leads to inconsistent and unpredictable changes in activity budgets. While we observed generalized trends in behavior, each gorilla’s response to the absence of zoo guests was varied, and despite observing specific trends in the troop’s overall activity budgets, these trends can be exaggerated by even a single member of the troop that responded strongly—either positively or negatively—to the absence of guests.

With only a single gorilla—the AM—showing statistically significant changes in one of the seven analyzed behaviors in the opposite direction of the rest of the troop, we did not find overwhelming evidence that zoo guests cause significant stress to gorillas in zoo-housed settings, in contrast to previous findings [27] that zoo visitors cause overt stress to troops. Our case report findings are consistent with the conclusions drawn from several other studies [14,24], that is, although there were some changes to behavioral patterns between the conditions, any findings cannot be generalized to suggest that zoo guests negatively or positively impact the welfare of gorillas in zoos. Our findings suggest that we must consider welfare at the individual level, as both intrinsic (sex, age, rearing status, and dominance rank in troop) and extrinsic (exhibit, weather, and visitor activity) factors uniquely influence each gorilla’s behavioral response. Demonstrating such patterns continues to be challenging, due to small sample sizes and inherent individual variation.

Though we did not find consistent and significant differences in behavior, we note that this case study is based on only one group of gorillas with limited data collection during an unprecedented pandemic. Consequently, we must consider the limitations of our study. First, the gorillas were given access to their off-exhibit holding areas on certain days by the exhibit staff whilst the zoo was closed to the public. We were unable to collect behavioral data whilst the gorillas were in holding, which may have impacted our results. Second, we treated zoo visitors as a binary variable (visitors were either in attendance or not in attendance). The problem with this approach has been highlighted by previous authors [24,27]. To provide more detailed information on the impact of zoo guests, future visitor effect studies must better quantify guest activity levels and interactions with gorillas as both initiators of and responders to gorilla behavior. Finally, our sample size was small. Even if each individual gorilla is considered as an independent data point (rather than the group as a single data point), we lack the power with six individuals to draw firm conclusions. For example, although 5 out of 6 gorillas showed decreases in foraging, we would have needed a larger group size, with 7 of 8 gorillas showing changes in the same direction to achieve statistical significance. We note that the behavior of the AM was consistently and noticeably different than that of the rest of the troop. Had we not adjusted our analyses for multiple comparisons, we would have seen significant changes in four behaviors, with notable increases in foraging and locomotion, and decreases in inactivity and autogrooming.

Other potential factors that could have influenced individual behavior include personality and/or exhibit design. These variables are both complex and difficult to measure. Gorillas’ personality types could have some potential impact on how they react to visitors [14]. For example, animals with more dominant personalities might respond differently to visitor changes than those with subordinate personalities. This could potentially be related to the unique behavior changes we saw in the AM, the most dominant gorilla in the troop. Exhibit design may also influence behavioral responses. The exhibit in our study was indoors with glass separating the gorillas from the visitors, allowing visitors to be in very close proximity to the gorillas which could have influenced their behavior. Each zoo has a different exhibit design, some include more space and vegetation to hide or a large moat offering significant spatial separation between gorillas and visitors. If our study were to be replicated at another zoo, these exhibit design factors would need to be taken into consideration.

In November of 2020, Buffalo NY was once again designated as an “orange zone” due to a rise in COVID-19 cases. This designation imposed restrictions on all non-essential businesses, including the Buffalo Zoo. Restrictions kept all of the indoor exhibits at the zoo closed to the public for several months, during which our team continued to collect behavior data. In the future we hope to again analyze the data from the second closure period to get a more complete picture of the gorilla’s response to a lack of visitors.

Further examination of the characteristics and behaviors of zoo visitors is likely to be an important area for future investigation. We would like to see if the visitors’ behavior can influence the gorillas’ behavior, as other taxa have been observed reacting according to specific visitor characteristics [28]. For example, some visitors passively observe the gorillas while others are loud and attempt to get the animals’ attention. We have observed visitors doing things such as knocking on the glass or beating their chest to provoke some sort of behavior change from the gorillas. By taking visitor behavior into account, we may be able to discover more specific details about visitor effect in gorillas.

We recognize the importance of visitor effect studies for the improvement of species-specific welfare standards that continuously evolve as we learn more about how animals in managed care interact with humans. In order to best serve their animal collections, we believe that zoological institutions should strive to permit a greater degree of animal choice within exhibit spaces. Studies such as these, that explore the interaction between human and non-human animals in the zoo, may inform future exhibit design and husbandry practices.

Considering the fact that all zoos offer unique environments, we encourage other zoos to conduct similar research if they are presented the opportunity to have exhibits closed to the public. A multi-institutional study could provide a more extensive look at how gorillas respond to visitors and possibly help us better understand what other factors such as crowd noise or behavior, exhibit design, or troop composition may influence gorilla responses.

## 5. Conclusions

We found that there were no consistent, significant changes in the gorilla’s behavior between 2019 and 2020. The small changes in behavior that we saw were not always consistent across the whole troop and few were statistically significant. We did see a slight overall decrease in autogrooming from all gorillas but it was not a significant difference. Of the other behaviors we focused on, there was no clear pattern of increase or decrease across the whole troop. For some individuals certain behaviors declined while the same behavior increased in other individuals. The most notable changes were in the behavior of the AM, who may have been less attentive to activities outside of the enclosure when visitors were absent. The findings of this case report highlight the importance of collecting routine data in order to facilitate comparison when unpredictable events occur. Such information could could shed light on the ways in which zoo guests may or may not impact the behavior and welfare of zoo collections.

## Figures and Tables

**Table 1:** Subjects for study and number of observations per subject during the 2019 and 2020 sampling periods. AM = adult male, AF = adult female, SAF = subadult female, JF = juvenile female, JM = juvenile male. Observations in which the focal subject was out of view for more than half the scans was not included in analyses. The table reflects the number of observations used out of the total number of observation sessions collected.

Individual	DOB	Sex	Observations Used/Total 2019	Observations Used/Total 2020
Koga (AM)	14 Aug 1987	M	17/21	20/27
Sidney (AF1)	6 Apr 1997	F	18/20	20/25
Lily (AF2)	19 Dec 2000	F	19/20	21/22
Amari (SAF)	8 Oct 2010	F	17/21	19/24
Nyah (JF)	4 Sept 2013	F	17/25	20/25
Kayin (JM)	10 Jan 2016	M	16/18	20/25

**Table 2:** Ethogram of behaviors that was analyzed for the present study. Behaviors that were excluded from analyses included those that were rare (comprising <5% of the activity budget), and those that were not relevant to the current study (i.e., orient to humans when no visitors were present) and have been omitted here for clarity.

State Behavior Analyzed for This Study	
Inactive	Sitting, standing, or laying while not engaged in physical activity
Locomotion	Movement on the ground terrestrially (T) or arboreally (A) utilizing Sclimbing structures in the exhibit
Auto-groom	Scratching, itching, rubbing, or self-grooming
R/R	Regurgitation and reingestion seen when a gorilla regurgitates food and consumes it again
Pluck	Pulling hair out
Social play	Any number of chasing, pulling, or biting contributing to ruff and tumble play
Forage	Searching or eating substrate
Out of view	The subject gorilla is beyond the observer’s field of view

**Table 3:** *p* values for Mann–Whitney U tests for each behavior and individual gorilla. *p* values adjusted for multiple comparisons (alpha = 0.01). Significant behaviors are noted with *.

Individual	Behavior						
	Forage	Inactive	Locomote	R/R	Auto-Groom	Social Play	Pluck
Koga (AM)	0.0585	0.02348	0.0319	n/a	0.0095 *	n/a	0.1863
Sidney (AF1)	0.2259	0.7636	0.247	0.6546	0.7982	0.3531	0.3531
Lily (AF2)	0.1011	0.3125	0.9373	0.8598	0.0653	0.3028	0.3028
Amari (SAF)	0.0292	0.01003 *	0.4245	n/a	0.9375	n/a	0.0795
Nyah (JF)	0.0849	0.3286	0.4321	n/a	0.5664	0.3222	n/a
Kayin (JM)	0.2691	0.08796	0.2262	n/a	0.5036	0.59	n/a

## Data Availability

The datasets analysed during the current study are available from the corresponding author on reasonable request.

## References

[1] Maple T., Perdue B.M. (2013). Zoo Animal Welfare.

[2] Ward S.J., Sherwin S., Clark F.E. (2018). Advances in applied zoo animal welfare science. J. Appl. Anim. Welf. Sci..

[3] Hill S.P., Broom D.M. (2009). Measuring zoo animal welfare: Theory and practice. Zoo Biol..

[4] Hosey G.R. (2000). Zoo animals and their human audiences: What is the visitor effect?. Anim. Welf..

[5] Davey G. (2007). Visitors’ effects on the welfare of animals in the zoo: A review. J. Appl. Anim. Welf. Sci..

[6] Stevens J.M.G., Thyssen A., Laevens H., Vervaecke H. (2013). The influence of zoo visitor numbers on the behaviour of harbour seals (*Phoca vitulina*). J. Zoo Aquar. Res..

[7] Larsen M.J., Sherwin S.L., Rault J. (2014). Number of nearby visitors and noise level affect vigilance in captive koalas. Appl. Anim. Behav. Sci..

[8] Thicks S. (2008). Is there a visitor effect on Abyssinian ground hornbills (*Bucorvus abyssinicus*), Papuan wreathed hornbills (*Aceros plicatus*), wrinkled hornbills (*Aceros corrugates*) and toco tucans (*Ramphastos toco*) in a captive zoo environment?. Plymouth Stud. Sci..

[9] Sherwin S.L., Harvey T.J., Magrath M.J.L., Butler K.L., Fanson K.V., Hemsworth P.H. (2015). Effects of visual contact with zoo visitors on black-capped capuchin welfare. Appl. Anim. Behav. Sci..

[10] López-Álvarez J., Sanjorge Y., Soloaga S., Crailsheim D., Llorente M. (2019). Looking for visitor’s effect in sanctuaries: Implications of guided visitor groups on the behavior of the chimpanzees at Fundació Mona. Animals.

[11] Hansen B.K., Hopper L.M., Fultz A.L., Ross S.R. (2020). Understanding the behavior of sanctuary-housed chimpanzees during public programs. Anthrozoös.

[12] Choo Y., Todd P.A., Li D. (2011). Visitor effects on zoo orangutans in two novel, naturalistic enclosures. Appl. Anim. Behav. Sci..

[13] Cooke C.M., Schillace M.A. (2007). Behavioral responses to the zoo environment by white handed gibbons. Appl. Anim. Behav. Sci..

[14] Stoinski T.S., Jaicks H.F., Drayton L.A. (2012). Visitor effects on the behavior of captive western lowland gorillas: The importance of individual differences in examining welfare. Zoo Biol..

[15] Blaney E.C., Wells D.L. (2004). The influence of a camouflage net barrier on the behaviour, welfare and public perceptions of zoo-housed gorillas. Anim. Welf..

[16] Lenczewski M., Halfdanardottir M.R., Margulis S. (2017). Now you see me (Now you don’t): A visual barrier study on a zoo-housed group of western lowland gorillas. Artic. Bones Discourse.

[17] Clark F.E., Fitzpatrick M., Hartley A., King A.J., Lee T., Routh A., Walker S.L., George K. (2012). Relationship between behavior, adrenal activity, and environment in zoo-housed Western lowland gorillas (*Gorilla gorilla gorilla*). Zoo Biol..

[18] Wood W. (1998). Interactions among environmental enrichment, viewing crowds, and zoo chimpanzees (*Pan troglodytes*). Zoo Biol..

[19] Wells D.L. (2005). A note on the influence of visitors on the behaviour and welfare of zoo-housed gorillas. Appl. Anim. Behav. Sci..

[20] Chamove A.S., Hosey G.R., Schaetzel P. (1988). Visitors excite primates in zoos. Zoo Biol..

[21] Lewis R.N., Chang Y., Ferguson A., Lee T., Clifforde L., Abeyesinghe S.M. (2020). The effect of visitors on the behavior of zoo-housed western lowland gorillas (*Gorilla gorilla gorilla*). Zoo Biol..

[22] Carder G., Semple S. (2008). Visitor effects on anxiety in two captive groups of western lowland gorillas. Appl. Anim. Behav. Sci..

[23] Davis N., Schaffner C.M., Smith T.E. (2005). Evidence that zoo visitors influence HPA activity in spider monkeys (Ateles geoffroyii rufiventris). Appl. Anim. Behav. Sci..

[24] Kuhar C.W. (2008). Group differences in captive gorillas’ reaction to large crowds. Appl. Anim. Behav. Sci..

[25] Altmann J. (1974). Observational study of behavior: Sampling methods. Behaviour.

[26] R Core Team R: A Language and Environment for Statistical Computing. R Foundation for Statistical Computing, Vienna, Austria. https://www.R-project.org/.

[27] Hosey G.R. (2005). How does the zoo environment affect the behaviour of captive primates?. Appl. Anim. Behav. Sci..

[28] Woolway E.E., Goodenough A.E. (2017). Effects of visitor numbers on captive European red squirrels (*Sciurus vulgaris*) and impacts on visitor experience. Zoo Biol..

